# Teaching and Learning in Times of COVID-19: Uses of Digital Technologies During School Lockdowns

**DOI:** 10.3389/fpsyg.2021.656776

**Published:** 2021-04-29

**Authors:** Juan-Ignacio Pozo, María-Puy Pérez Echeverría, Beatriz Cabellos, Daniel L. Sánchez

**Affiliations:** Department of Basic Psychology, Faculty of Psychology, Autonomous University of Madrid, Madrid, Spain

**Keywords:** digital technologies uses, constructive learning, reproductive learning, learning and teaching conceptions, learning outcomes, COVID-19

## Abstract

The closure of schools as a result of COVID-19 has been a critical global incident from which to rethink how education works in all our countries. Among the many changes generated by this crisis, all teaching became mediated by digital technologies. This paper intends to analyze the activities carried out during this time through digital technologies and the conceptions of teaching and learning that they reflect. We designed a Likert-type online questionnaire to measure the frequency of teaching activities. It was answered by 1,403 teachers from Spain (734 primary and 669 secondary education teachers). The proposed activities varied depending on the learning promoted (reproductive or constructive), the learning outcomes (verbal, procedural, or attitudinal), the type of assessment to which the activities were directed, and the presence of cooperative activities. The major result of this study was that teachers used reproductive activities more frequently than constructive ones. We also found that most activities were those favoring verbal and attitudinal learning. The cooperative activities were the least frequent. Finally, through a cluster analysis, we identified four teaching profiles depending on the frequency and type of digital technologies use: Passive, Active, Reproductive, and Interpretative. The variable that produced the most consistent differences was previous digital technologies use These results show that Information and Communication Technologies (ICT) uses are reproductive rather than constructive, which impedes effective digital technologies integration into the curriculum so that students gain 21st-century competencies.

## Introduction

When schools were closed in most countries in March 2020 because of the COVID-19 pandemic, teachers had no other option but to change their classrooms into online learning spaces. It was a critical global incident. In research on identity and teacher training (Tripp, 1993; Butterfield et al., 2005; Monereo, 2010), a critical incident is an unexpected situation that hinders the development of the planned activity and that, by exceeding a certain emotional threshold, puts the identity in crisis and obliges that teachers review their concepts, strategies, and feelings. Thus, these incidents can become meaningful resources for training and changing teaching and learning practices because they allow us to review our deep beliefs (Monereo et al., 2015).

The critical global incident generated by the pandemic forced most teachers to assume virtual teaching where they had to use digital technologies, sometimes for the first time, to facilitate their students’ learning. The closure of schools as a consequence of COVID-19 led to substantial changes in education with profound consequences. Today we know that educational inequalities have widened (Dorn et al., 2020), while students have suffered greater social and emotional imbalances (Colao et al., 2020). In this context, families have also been more involved in the school education of their children (Bubb and Jones, 2020). Moreover, concerning the objectives of this study, it has been necessary to rethink the teaching strategies in the new virtual classrooms. In fact, this research focuses precisely on analyzing the uses that teachers made of the digital technologies or Information and Communication Technologies (ICT) (from now on, we will use this acronym) during the confinement to become familiar with their practices and use them to review their conceptions of teaching and learning.

For several decades, many authors have argued that ICT as educational devices facilitate the adaptation of teaching to each student. Some argue this is because they can promote collaboration, interactivity, the use of multimedia codes, and greater control of learning by the learner (e.g., Jaffee, 1997; Collins and Halverson, 2009). In this way, their integration in the curriculum would contribute to the acquisition of 21st-century competencies (autonomy, collaboration, critical thinking, and problem-solving) that the OECD (Ananiadou and Claro, 2009) links to the so-called “global competence” that should define the current education (Ertmer et al., 2015).

However, after decades of use of ICT in classrooms, they have not fully achieved their promise to transform teaching and learning processes. The results of a lot of international studies are, in fact, quite discouraging, like those claimed by the PISA studies (OECD, 2015). In its report, the OECD(2015, p. 3) concludes that “the results also show no appreciable improvements in student achievement in reading, mathematics or science in the countries that had invested heavily in ICT for education.” Thus, Biagi and Loi (2013) found that the more education ICT uses reported, the less learning in reading, mathematics, and science achieved. These data caused even Andreas Schleicher, head and coordinator of PISA studies, to claim that “the reality is that technology is doing more harm than good in our schools today” (Bagshaw, 2016).

These conclusions contrast with the results obtained in most of the experimental research on the effects of ICT on learning. A decade ago, after conducting a second-order meta-analysis of 25 meta-analyses, Tamim et al.(2011, p. 14) found “a significant positive small to moderate effect size favoring the utilization of technology in the experimental condition over more traditional instruction (i.e., technology-free) in the control group,” a conclusion that is still valid today. Various studies and meta-analyses reflect moderate but positive effects on learning, whether for example from the use of touch screens in preschools (Xie et al., 2018), from cell phones (Alrasheedi et al., 2015; Sung et al., 2015) or video games (Clark et al., 2016; Mayer, 2019). It has also been found that they favor collaboration in secondary education (Corcelles Seuba and Castelló, 2015) or learning mathematics (Li and Ma, 2010; Genlott and Grönlund, 2016), science (Hennessy et al., 2007) or second languages (Farías et al., 2010).

What is the reason for this disagreement between research conducted in experimental laboratories and large-scale studies? Many factors could explain this distance (de Aldama, 2020). But one difference is that the experimental studies have been carefully designed and controlled to promote these forms of learning mentioned above, while the usual work in the classroom is mediated by the activity of teachers who, in most cases, have little training using ICT (Sigalés et al., 2008). Several authors (Gorder, 2008; Comi et al., 2017; Tondeur et al., 2017) conclude that it is not the ICT themselves that can transform the classroom and learning, but rather the use that teachers make of them. While the experimental studies mostly promote activities that encourage autonomous learning (Collins and Halverson, 2009), the most widespread uses of ICT, as reflected in these international studies with more diverse samples, report other kinds of use whose benefits are more doubtful.

Different classifications of teachers’ use of ICT in the classroom have been proposed in recent years (e.g., Gorder, 2008; Mama and Hennessy, 2013; Comi et al., 2017). Tondeur et al. (2008a) differentiate three types of educational computer use: (a) basic computer skills; (b) use of computers as an information tool, and (c) use of them as a learning tool. Laying aside the acquisition of basic skills related to digital devices, learning is promoted by the last two uses that lead to second-order digital skills related to information management and its conversion into knowledge (Fulton, 1997; Gorder, 2008). Thus, the distinction is usually made between two types of use. The first use is aimed at traditional teaching, focused on the transmission and access to information, and usually called teacher-centered use (although perhaps it should be called content-centered use). The second one, called student-centered use, promotes diverse competencies (autonomy, collaboration, critical thinking, argumentation, and problem-solving) and is part of the Global Competence characteristic of 21st-century education (Ananiadou and Claro, 2009; OECD, 2019, 2020). According to Tondeur et al. (2017), integration of ICT in education requires assuming a constructivist conception of learning and adopting a student-centered approach in which the students manage the information through the ICT instead of, as in the more traditional approach (content-centered), it being the teacher who uses the ICT.

The experimental studies mentioned above show that student-centered approaches improve verbal earning, producing a better understanding of the subjects studied, promoting self-regulation of the learning processes themselves, and generating critical and collaborative attitudes toward knowledge. Thus, Comi et al.(2017, pp 36–37), after analyzing data from different standardized assessments, conclude: “computer-based teaching practices increase student performance if they are aimed at increasing students’ awareness of ICT use and at improving their navigation critical skills, developing students’ ability to distinguish between relevant and irrelevant material and to access, locate, extract, evaluate, and organize digital information.” Besides, they also found a slight negative correlation between using ICT to convey information and academic performance.

In spite of these better results of adopting student-centered uses, the studies support that the most frequent uses in classrooms are still centered on the teachers, who indeed use ICT as a substitute for other more traditional resources to transmit information (Loveless and Dore, 2002; Sigalés et al., 2008; de Aldama and Pozo, 2016). Even if what Ertmer (1999) called type I barriers are overcome, related to the availability of these technological resources and the working conditions in the centers, several studies show that there are other types II barriers that limit the use of ICT (Ertmer et al., 2015); in particular, the conceptions about learning and teaching to the extent that they mediate the use of ICT (Hermans et al., 2008).

Different studies have shown that these teachers’ beliefs about learning and teaching are the best predictor of the use made of ICT in the classroom (Ertmer, 2005; Ertmer et al., 2015). Most of the work on these beliefs (Hofer and Pintrich, 1997, 2002; Pozo et al., 2006; Fives and Gill, 2015) identifies two types of conceptions: some closer to a reproductive vision of learning, which would be related to the teacher or content-centered teaching uses, and others nearer to constructivist perspectives, which promote student-centered teaching uses. Studies show teachers who have constructivist beliefs tend to use more ICT than those with more traditional beliefs (Judson, 2006; Law and Chow, 2008; Ertmer et al., 2015). They also employ them in a more student-centered way, and their uses are oriented toward the development of problem-solving skills (Tondeur et al., 2017). On the other hand, teachers with more traditional beliefs use them primarily to present information (Ertmer et al., 2012).

However, the relationship between conceptions and educational practices is not so clear and linear (Liu, 2011; Fives and Buehl, 2012; Tsai and Chai, 2012; Mama and Hennessy, 2013; Ertmer et al., 2015; de Aldama and Pozo, 2016; de Aldama, 2020). Many studies show a mismatch between beliefs and practices, above all, when we refer to beliefs closer to constructivism that do not always correspond to constructive or student-centered practices. We can distinguish three types of arguments that explain the mismatches. First, the beliefs seem to be more complex and less dichotomous than what is assumed (Ertmer et al., 2015). The studies comparing beliefs and practices tend to focus on the more extreme positions of the spectrum -reproductive vs. constructive beliefs-, despite research showing they are part of a continuum of intermediate beliefs between both aspects (Hofer and Pintrich, 1997, 2002; Pérez Echeverría et al., 2006). Thus, for example, the so-called interpretive beliefs maintain traditional reproductive epistemological positions. People who have these conceptions think that learning is an exact reflection of reality or the content which should be learned, whereas they also think teaching is mediated by cognitive processes of the learner which are based on his or her activity (Pozo et al., 2006; López-Íñiguez and Pozo, 2014; Martín et al., 2014; Pérez Echeverría, in press). Other examples of this belief can be found in the technological-reproductive conception described by Strauss and Shilony (1994), which is close to a naïve information processing theory.

Second, we must acknowledge that neither teachers’ beliefs nor their educational practices remain stable but vary according to the teaching contexts. As Ertmer et al. (2015) claim, beliefs are not unidimensional, but teachers assume them in varying degrees and with different types of relationships. The teacher’s beliefs seem to be organized in profiles that gather aspects of the different theories about teaching and whose activation depends on the contextual demands (Tondeur et al., 2008a; Bautista et al., 2010; López-Íñiguez et al., 2014; Ertmer et al., 2015).

Third, we consider that this multidimensionality of beliefs makes them very difficult to measure or evaluate (Pajares, 1992(Schraw and Olafson, 2015; see also Ertmer et al., 2015; Pérez Echeverría and Pozo, in press), so perhaps different studies are measuring different components. For example, many studies focus on explicit beliefs, or “what teachers believe to be true” for learning, and therefore evaluate more the general ideas about what ICT-based education should be. Usually, these statements tend to be relatively more favorable to the advantages mentioned above. In this paper, we have chosen to analyze teachers’ stated practices as a means of addressing specific beliefs about teaching.

In addition to beliefs, other variables have been identified that influence the use of ICTs such as gender, age, educational level, or subject curriculum, with results that are usually inconclusive. Thus, while Mathews and Guarino (2000) found that men were more inclined toward the use of ICTs than women, in other studies no differences were found (Gorder, 2008; Law and Chow, 2008). Similarly, other studies (van Braak et al., 2004; Suárez et al., 2012) concluded that there was an inverse relationship between the age of the teachers and their interest in ICT, but other studies did not confirm this conclusion (Gorder, 2008; Law and Chow, 2008; Inan and Lowther, 2010). Finally, the teaching experience gives equally ambiguous results; some papers report a negative relationship (Mathews and Guarino, 2000; Baek et al., 2008; Inan and Lowther, 2010) while others find no relationship (Gorder, 2008).

The influence of factors like educational level or curriculum subjects has also been analyzed. The data seem to be more conclusive regarding educational level: teachers in secondary education have more favorable attitudes toward ICT than teachers of earlier levels (Gorder, 2008; Vanderlinde et al., 2010). However, the data are not so conclusive regarding the influence of curriculum subjects (Williams et al., 2000; Gorder, 2008; Vanderlinde et al., 2010).

Although it will take time to understand what has happened in teaching during these months, many studies and proposals have analyzed the use of ICT in distance education. We can classify them into three types of research. The first type of analyses has measured the impact of classroom closures on the education of students, many of them focusing on their effects on inequality or the way different countries have dealt with this crisis (Crawford et al., 2020; Reimers and Schleicher, 2020; Zhang et al., 2020). Second, studies have aimed at proposing principles that should guide the use of ICT in the classroom (Ferdig et al., 2020; Rapanta et al., 2020; Sangrà et al., 2020). The last ones, which are close to the aims of this study, are focused on how teachers have used ICT for the COVID-19 crisis. Some of these studies have carried out qualitative case analyses in different contexts, institutions (Koçoğlu and Tekdal, 2020; Rasmitadila et al., 2020), and even countries (Hall et al., 2020; Iivari et al., 2020). However, others have resorted to the use of questionnaires applied to larger samples to inquire about the teaching experience for confined education (Devitt et al., 2020; Luengo and Manso, 2020; Tartavulea et al., 2020; Trujillo-Sáez et al., 2020). These studies have concluded the most common use by teachers was to upload materials to a platform (Tartavulea et al., 2020); the most activities were teacher-centered (Koçoğlu and Tekdal, 2020); or the more constructivist the teachers are, the more ICT use is reported for confined education (Luengo and Manso, 2020).

However, despite these indications, there has been no study that analyzes the activities and uses of ICT in school during confinement. What learning have teachers prioritized in this period? Has it been more oriented toward verbal, procedural, or attitudinal learning? (Pozo, in press). Through what activities, either more constructive or reproductive, have these learnings been promoted? Have the ICT been used to assess the accumulation of information or the global competencies in its management? What variables prompt carrying out one type of activity or another? These are some questions that have guided our research and are reflected in the following specific objectives.

1. Identifying the frequency with which Spanish teachers of primary, and compulsory and non-compulsory secondary education carried out activities using ICT during the pandemic, and how some variables influence this frequency (gender, teaching experience, previous ICT use, educational level, and curriculum subjects).
2. Analyzing the type of learning (reproductive or teacher-centered vs. constructive or student-centered) promoted most frequently by these teachers, as well as the influence of the variables mentioned.
3. Analyzing the types of outcomes (verbal learning, procedural learning, or attitudinal learning), assessment, and social organization promoted by the ICT and the possible influence of the mentioned variables.
4. Investigating if different teaching profiles can be identified in the use of ICT, as well as their relationship with the variables studied.

Regarding objective 1, as the contradictory results reviewed in the Introduction showed, it is difficult to sustain a concrete hypothesis. However, in the case of objective 2, as argued in the Introduction, we expect to find a higher frequency of reproductive activities (or teacher-centered) than constructive (student-centered). Along the same lines, concerning the third objective, we hope to find more activities oriented to verbal learning, reproductive assessment, and individual organization of tasks, with few activities based on cooperation between students. Finally, about objective 4, we hope to identify teacher profiles that differ in the frequency and type of activities proposed to their students and that these profiles are related to some of the demographic variables analyzed in the study.

## Materials and Methods

### Task and Procedure

To achieve these objectives, we designed a questionnaire on ICT through the Qualtrics software and sent telematically to various networks of teachers and primary and secondary education centers in Spain. For the construction of the questionnaire, we consulted different blogs where teachers shared the activities they were applying during the pandemic. The questionnaire was composed of two parts. In the first one, after participants gave informed consent, they were requested to provide personal and professional information (see Table 2). The second part comprised 36 items that described different types of teaching activities. Participants were asked to rate how often they carried them out on a Likert scale (1, Never; 2 Some days per month; 3, Some days per week; and 4, Every day). After the analysis of the methodologies carried out in the Introduction, we considered asking teachers what they were doing in their classrooms was the most accurate procedure to know the true practices they were carrying out. On the one hand, we wanted to avoid the bias of classic questionaries on conceptions that require teachers to express their agreement with some beliefs. On the other hand, the analysis of teachers’ actual practices in their classrooms would require a different, more qualitative work, with a smaller sample size.

As we show in Table 1, these activities were directed toward reproductive and constructive learning and different types of learning outcomes (verbal, procedural, and attitudinal), assessment (usually called summative and formative assessment), and cooperative activities.

**TABLE 1:** Structure and examples of questionnaire items.

		Reproductive		Constructive

Dimension	*N*	Example of items	*N*	Example of items
Verbal learning	4	Item 1. I record a presentation explaining a topic and upload it to a platform so that my students can see it and study it when they need to.	4	Item 5. I send different materials about a topic to my students, so they can develop their point of view and reflect it in a task.
Procedural learning	4	Item 9. I record the instructions about how to do a task and upload them to a platform for my students to put them into practice.	4	Item 13. I present to my students an open problem so that they can plan an investigation or project themselves and carry it out.
Attitudinal learning	4	Item 17. I promote in my students the habit of following a fixed schedule for classes and activities.	4	Item 21. We decide as a group which behavior patterns we are going to follow to better manage communication in online classes.
Assessment	4	Item 29. I send questions or exercises for the students to complete, and then I send them the correct answers so that by seeing their mistakes, they know what to correct.	4	Item 33. I give students answers from other classmates about tasks they have all done so that they can evaluate them and justify how they could improve.

**TABLE 2:** Characteristics of the sample and variables.

Variable	Category	N
Gender	Men	405
	Women	992
	Others	6
Teaching experience	5 years or fewer	321
	From 6 to 15 years	396
	From 16 to 25 years	422
	26 years or more	264
Educational level	1st, 2nd, or 3rd of primary education (6–9 years) or First years of primary education	348
	4th, 5th, or 6th of primary education (9–12 years) or Last years of primary education	386
	Compulsory secondary education (12–16 years)	499
	Non-compulsory secondary education (12–18 years)	170
Primary curriculum subjects	Generalists	421
	Specialists	302
	Others	11
Secondary curriculum subjects	Spanish language	92
	Mathematics	82
	Social sciences	80
	Natural sciences	109
	Foreign language	86
	Physical education	31
	Technology	59
	Others	130
Previous ICT use	Never	380
	Some days per month	572
	Some days per week	297
	Every day	154

### Participants

The participants were primary and secondary education teachers who were working in Spain when they completed the questionnaire. In Spain, compulsory education is from 6 to 16 years. In primary education (6–12 years), a single generalist teacher imparts most of the subjects, while specialist teachers (music, physical education, foreign language, etc.) only attend class during the hours of their subjects. After compulsory secondary education, there is a non-compulsory secondary education (16–18 years old) that is taught in the same centers as compulsory secondary education and by the same teachers.

We used directories of emails from public, private schools, and high schools of Spain to get in contact with the participants. Besides, to encourage participation, we raffled 75 euros for the purchase of teaching materials among all participants. We collected 1,541 answers. We eliminated 52 of them because they belonged to people who were not teachers of primary or secondary education in Spain. Then, we removed 45 participants who completed the questionnaire in less than 5 min, insufficient time to read and complete it, and we excluded 41 participants who indicated the 3rd (“Some days per week”) or 4th option (“Every day”) in over 80% of the items. We argue this exclusion as it is unlikely that a teacher could carry out such a quantity of activities in the span of a week. The questionnaire has 36 activities, so doing over 80% of items with a frequency of a minimum some days per week implies carrying out almost 29 activities per week. We consider this is not possible in the pertaining virtual class context and noted several contradictions in the answers. Therefore, the final sample had 1,403 teachers (see Table 2). Note that the sum of all variables does not reach this total because some values were so unusual that they were not considered in the statistical analyses.

### Data Analysis

To ensure the consistency of the questionnaire and the dimensions, a reliability analysis was carried out using Cronbach’s Alpha coefficient. The reliability of the scale was 0.90, the reproductive and constructive scales obtained alphas above 0.75, and the verbal, procedural, attitudinal, assessment, and cooperation dimensions got alphas above 0.65.

The 1, 2, and 3 objectives were analyzed with one and two-factor ANOVA. These factors can be both repeated measures and completely randomized, according to the characteristics of the variable. Besides, we carried out *post hoc* analysis in which the Tukey or Bonferroni correction was applied depending on whether the ANOVA was 1 or 2 factors, to see the differences between categories in the ANOVA analyses. However, *post hoc* analyses were only performed on the ANOVA of the two factors when the interaction effects were significant.

Finally, a cluster analysis was implemented to identify different teaching profiles (objective 4). Once identified, we created contingency tables and their corresponding Corrected Typified Residuals (CTR) to know which variables were related to each profile. Finally, we carried out ANOVA to analyze the differences between profiles according to each of the designed dimensions. All the statistical analyses were carried out using SPPS version 26.

## Results

The results are written referring to what the teachers were doing to facilitate reading. However, in all cases, we refer to declared activities.

### Frequency of Activities Carried Out

Regarding the first objective, teachers performed the activities between Some days per week and Some days per month on average (*M* = 2.44, *SD* = 0.50). However, this frequency varied according to teaching experience, educational level, curriculum subject, and previous ICT use. Gender did not produce differences (see Table 3). In the case of teaching experience, according to the *post hoc* tests, teachers with intermediate experience (from 16 to 25 years) carried out a lower number of activities than novice teachers (5 years or fewer) (*p* < 0.05). In turn, teachers who taught children between 6 and 9 years old were also less active than the rest (*p* < 0.01). Within primary education, the generalists, who spend more time with the same students, proposed more activities than the specialists (*p* < 0.01). In secondary education, the teachers of Spanish language were more active than those of mathematics and physical education (*p* < 0.01). Finally, there seems to be a positive linear relationship between previous ICT use and the amount of activity for confined education (*F* = 61.66, *p* < 0.001).

**TABLE 3:** Influence of personal and professional variables on the frequency of activities.

Variable	*F*	η_*p*_^2^
Gender	0.01	0.01
Teaching experience	2.62*	0.01
Educational level	7.60***	0.02
Primary curriculum subject	9.79 **	0.06
Secondary curriculum subject	4.35***	0.05
Previous ICT use	25.60***	0.05

*p < 0.05; **p < 0.01; and ***p < 0.001.

### Teaching Activities: Reproductive or Constructive?

Nevertheless, we were not so much interested in the total amount of activities carried out as in the type of learning they promoted (reproductive or constructive). For this, we proposed objective 2. The data was overwhelming. They showed much greater use of reproductive (*M* = 2.79, SD = 0.50) than constructive (*M* = 2.16, SD = 0.60) learning activities (*F* = 2,217.91, *p* < 0.001, η_*p*_^2^ = 0.61). This is the largest and most robust effect size in this study; it occurs in all groups and for all variables (*p* < 0.001), although to a different degree, as shown in Table 4.

**TABLE 4:** Influence of the different variables on the type of activity.

Variable	*F*	η_*p*_^2^
Gender	2.84	0.10
Teaching experience	4.87**	0.01
Educational level	3.64*	0.01
Primary curriculum subject	0.03	0.01
Secondary curriculum subject	7.05***	0.07
Previous ICT use	4.80***	^0.01^

*p < 05; **p < 0.01; and *** *p* < 0.001.

*Post hoc* results reveal that novice teachers (5 years or fewer), the most active group according to the previous analysis, performed more reproductive activities than teachers with experience from 16 to 25 years (*p* < 0.01), the least active one. However, the most experienced teachers (more than 25 years) executed more constructive activities than those with intermediate experience (from 16 to 25 years) (*p* < 0.05). The teachers of children between 6 and 9 years old did less reproductive and constructive activities (*p* < 0.05) than the rest of the groups, with significant differences in all cases except in the case of the teachers of non-compulsory secondary education, who stated less reproductive activities than they did.

In secondary education, the mathematics teachers did less constructive activities than those of Spanish language and social sciences (*p* < 0.05). In turn, physical education teachers performed less reproductive activities than the rest of their classmates (*p* < 0.01).

Finally, the higher the previous ICT the teachers used, the higher the frequencies indicated by them in both reproductive (*F* = 33.57, *p* < 0.001) and constructive activities (*F* = 61.61, *p* < 0.001). Notwithstanding, the size of the observed effect shows greater differences in the case of constructive activities (reproductive, *F* = 13.94, *p* < 0.001, η_*p*_^2^ = 0.29, vs. constructive, *F* = 25.60, *p* < 0.001, η_*p*_^2^ = 0.95).

### Learning Outcomes, Assessment, and Cooperation Dimensions

**FIGURE 1:**
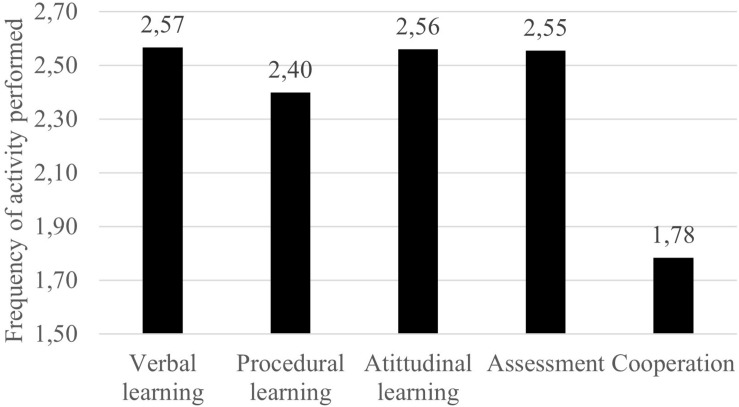
Average of the frequencies of each type of activity.

The third objective was to determine what kind of learning outcomes resulted from the activities. As we show in Figure 1, the teachers focused more on verbal and attitudinal learning than on procedural (*F* = 100.11, *p* < 0.001, η_*p*_^2^ = 0.07). On the other hand, the mean responses of the assessment tasks were similar to those of verbal learning and attitudinal learning, but the cooperative activities were less frequent than the remainder (*p* < 0.001), performed between never and some days per month (*M* = 1.78; SD = 0.74). However, as we see in Table 5, these results are mediated by the effect of some variables.

**TABLE 5:** Influence of different variables on the frequency of activities for each dimension.

	Two-factors ANOVA (Interaction effect)		One-factor ANOVA									
	Verbal, procedural, and attitudinal learning		Verbal learning		Procedural learning		Attitudinal learning		Assessment		Cooperation	
	*F*	η_*p*_^2^	*F*	η_*p*_^2^	*F*	η_*p*_^2^	*F*	η_*p*_^2^	*F*	η_*p*_^2^	*F*	η_*p*_^2^
Gender	57.74***	0.02	0.01	0.00	5.55*	0.00	14.31***	0.01	0.19	0.00	6.90**	0.01
Teaching experience	0.919	0.48	2.43	0.01	1.29	0.00	1.03	0.00	3.69*	0.01	1.69	0.00
Educational level	21.53***	0.04	5.23**	0.01	12.42***	0.03	8.29***	0.02	14.09***	0.03	8.74***	0.02
Primary curriculum subject	1.717	0.07	6.92***	0.05	7.16***	0.05	7.34***	0.05	16.60***	0.10	1.47	0.01
Secondary curriculum subject	9.89***	0.10	8.37***	0.09	1.09*	0.01	4.99***	0.05	11.31***	0.11	9.40***	0.10
Previous ICT use	4.70***	0.01	11.96***	0.03	30.08***	0.06	20.29***	0.04	6.18***	0.01	28.30***	0.06

*p < 0.05; **p < 0.01; ***p < 0.001.

*Post hoc* analyses show that men carried out more activities focused on procedural learning than women (*p* < 0.05), who in turn promoted more activities related to attitudinal learning (*p* < 0.001). Men also carried out more cooperation activities than women (*p* < 0.01), but there were no differences among them in the Assessment activities. However, the only effect related to teaching experience shows that less experienced teachers (5 years or fewer) carried out more assessment activities than teachers with intermediate experience (from 16 to 25 years) (*p* < 0.05).

The teachers of the youngest children (6–9 years old) carried out more activities aimed at attitudinal learning (*p* < 0.05) and fewer at procedural learning (*p* < 0.01) than the rest of the teachers. Interestingly, the activities aimed at attitudinal learning decreased progressively when the educational level increased, with differences between the upper level of primary education (9–12 years) and secondary education (*p* < 0.001). At the same time, the older the students were, the more verbal learning activities they performed, with differences between the first years of primary education (6–9 years) and secondary education (12–18) (*p* < 0.05). Besides, the assessment and cooperation activities became more frequent as the educational levels advanced, with differences in both cases between the teachers of the first years of primary education (*p* < 0.01) and the last years of primary education and non-compulsory secondary education (*p* < 0.05).

In secondary education, verbal learning predominates in almost every subject. However, the Spanish language and foreign language teachers also carried out many activities aimed at attitudinal learning. Only in technology were more activities aimed at procedural learning executed compared to the others (*p* < 0.05). At the same time, the mathematics teachers stand out for their little use of cooperation activities. To sum up, the activities aimed at verbal learning increase their frequency when the educational level increases, while attitudinal learning decreases. Nevertheless, the characteristics of each subject have some influence on the increases among educational levels. The cooperation activities also increase, although their frequency is still small. Finally, again, the higher the previous ICT use, the higher the frequency of all activities during the pandemic (*p* < 0.001).

**FIGURE 2:**
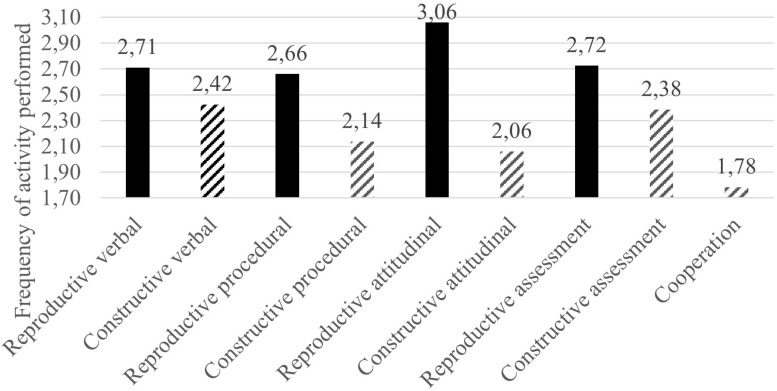
Average of the reproductive and constructive activities in each dimension.

But all these differences become more meaningful when we look at the type of learning (reproductive or constructive) that is promoted by these activities. Again, as we see in Figure 2, there is a considerable difference between the reproductive and constructive activities regardless of the dimension involved (see Table 6), a trend also confirmed by the low frequency of cooperation activities that, by their nature, promote constructive learning. It is remarkable that the highest differences between both scales happen in attitudinal learning. In fact, the most frequent activities in the questionnaire involved attitudinal reproductive learning.

**TABLE 6:** Differences between reproductive and constructive activities in the dimensions.

	Type of activity	*F*	η_*p*_^2^
Verbal learning	Reproductive	180.05***	0.11
	Constructive		
Procedural learning	Reproductive	675. 20***	0.33
	Constructive		
Attitudinal learning	Reproductive	2,908.24***	0.68
	Constructive		
Assessment	Reproductive	271.46***	0.16
	Constructive		

*p < 0.05; **p < 0.01; and ***p < 0.001.

### Profiles of Teachers in the Use of ICT

**FIGURE 3:**
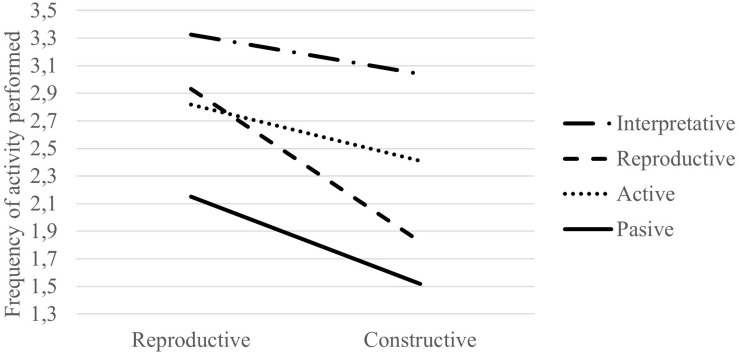
Frequency of use of reproductive and constructive activities for each teachers’ profile.

Our final objective was to identify possible profiles in the use of ICT during confined education. For this purpose, we proceeded with a cluster analysis that allowed us to identify different teaching profiles as we showed in Figure 3. After testing clusters of three centers in which the groups only differed in the number of activities, we executed a four centers cluster, which showed differences in the amount of activity (*F* = 2,220.33, *p* < 0.001, η_*p*_^2^ = 0.83) and the mean differences between reproductive and constructive activities (*F* = 310.39, *p* < 0.001, η_*p*_^2^ = 0.40).

• The first profile (“Passive”) was composed of 327 teachers who were characterized by a very low activity (MD = 0.63, SD = 0.02, *p* < 0.001), essentially reproductive (*M* = 2.15, SD = 0.35) and scarcely constructive (*M* = 1.52, SD = 0.29).
• The second profile (“Active”) was composed of 424 teachers, was the most numerous. It had a very similar pattern to the previous one, focused mainly on reproductive activities (*M* = 2.82, SD = 0.33) rather than constructive (*M* = 2.41, SD = 0.21) but with a higher level of activity (*MD* = 0.41, SD = 0.02, *p* < 0.001).
• The third profile (“Reproductive”) was composed of 263 teachers with a similar level of activity to the previous one. However, they have a relatively higher frequency of reproductive activities (*M* = 2.93, SD = 0.29) with hardly any constructive activities (*M* = 1.82, SD = 0.24).
• The fourth profile (“Interpretative”) which was composed of 389 teachers, was corresponded to the most active teachers. This profile had the smallest differences between reproductive (*M* = 3.32, SD = 0.29) and constructive activities (*M* = 3.04, SD = 0.31), (*MD* = 0.29, *SD* = 0.02, *p* < 0.001). According to the terminology used in the introduction, we have called it Interpretative because it integrated both types of activities.

**FIGURE 4:**
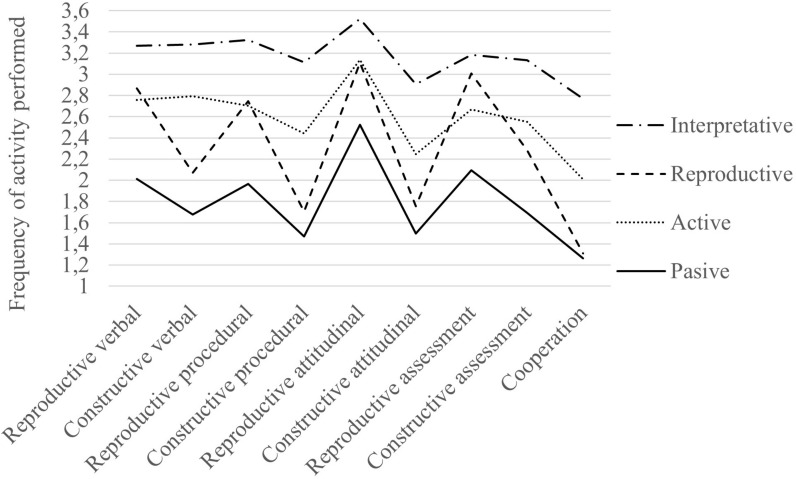
Use of each dimension for each teachers’ profile.

Among the different profiles, we found systematic differences in the dimensions and types of learning. In fact, all differences among profiles were significant (*p* < 0.01) except between the Active and Reproductive profiles in verbal, procedural, and attitudinal reproductive learning. There were also no differences between the Passive and Reproductive profiles in cooperative activities because of their low frequency in both groups. On the other hand, teachers in the Interpretive profile carried out more activities in all dimensions than the rest of the groups; the teachers of the Passive profile did fewer tasks than the others (except in the cases already indicated) and finally, the other two profiles maintained an intermediate level of activity, with the difference that the teachers of the Reproductive profile focused almost exclusively on reproductive activities as we see in Figure 4.

The distribution of teachers in each of the four profiles varied depending on educational level (χ^2^ = 29.57, *p* < 0.001), primary curriculum subjects (χ^2^ = 60.97, *p* < 0.001), secondary curriculum subjects (χ^2^ = 60.97, *p* < 0.001), and previous ICT use (χ^2^ = 77.46, *p* < 0.001). We did not find any relationship with gender or teaching experience, the variables with the least influence in the study.

As we see in Table 7, the first profile or Passive was over-represented by teachers of children aged 6–9, and teachers of non-compulsory secondary education were under-represented. Between the primary education teachers, specialists predominated, and there were practically no generalist teachers. The only secondary education teachers that appeared in this profile were physical education ones. Finally, there is a significant number of teachers who had not used ICT with their students before the confinement, and there was hardly any representation of those who had most used them.

**TABLE 7:** Variables related to each of the profiles.

	Profile 1	Profile 2	Profile 3	Profile 4
**Genre**				
Teaching experience	*5 years or fewer^1^, CTR* = −*2.1 (19%^2^)* From 16 to 25 years, CTR = 2.3 (27.3%)			
Educational level	*Non-compulsory secondary, CTR* = −*2.4 (15.9%).* From 6 to 9 years, CTR = 5 (33%)			*From 6 to 9 years CTR* = −*2.3 (14.7%)*
Primary curriculum subjects	*Specialists, CTR* = *5.3 (37.1%)* Generalists, *CTR* = −5.3 (19.5%)			*Specialists* = −*3.6 (11.6%)* Generalists, *CTR* = 3.6 (22.1%)
Secondary curriculum subjects	Physical Education, *CTR* = 3.6 (41.9%)	*Foreign Language, CTR* = −*2.7 (18.6%)* Spanish Language, *CTR* = 2.1 (40.2%)	*Mathematics, CTR* = −*3.1 (46.3%)*	Spanish Language, *CTR* = 2 (27.2%) Mathematics, *CTR* = −2.5 (9.86%)
Previous ICT use	*Every day, CTR* = −*3.2 (13%). Some days per week, CTR* = −*2.2 (18.5%)* Never, *CTR* = 3.00 (28.9%)		Some days per week, *CTR* = −2.7 (21.5%) Never, *CTR* = 3.7 (35%)	*Never, CTR* = −*6 (8.4%)* Some days per week, *CTR* = 4.9 (28.6%) Every day, *CTR* = 3.7 (29.9%)

*^1^Categories written in italics under-represent the expected value in the population. The rest of the categories overrepresent the expected value. ^2^It refers to the percentage of a category that is distributed among the different profiles.*

The second or Active profile is distributed homogeneously way among the different educational levels. It is predominantly formed by secondary education teachers of Spanish language and social sciences. In the third or Reproductive profile, secondary education teachers who taught mathematics, and those who had never used ITC in the classroom were over-represented.

The fourth or Interpretative profile, characterized by integrating reproductive and constructive activities, had hardly any teachers of children from 6 to 9 years old nor specialist teachers of primary education, unlike the first profile. However, this profile included a high number of generalist teachers of primary education and Spanish language teachers of secondary education. On the other hand, it had a few mathematics teachers from secondary education who were over-represented in the Reproductive profile. Finally, the teachers who used ICT more before confinement were also over-represented, and there were hardly any teachers who had not used them.

## Discussion and Conclusion

In this study, taking advantage of the critical incident caused by the COVID-19 pandemic, we analyzed the type of activities with ICT that primary and secondary education teachers proposed to their students. Our purpose was to check if, in this context, ICT contributed to promoting more constructive ways of teaching. The most dominant effect of the results, related to the second aim of the study, showed that teachers carried out significantly more activities oriented to reproductive learning than constructive ones. In other words, they preferred teacher-centered activities to student-centered ones. This effect was very robust (*F* = 2,217.91, *p* < 0.001, η_*p*_^2^ = 0.61), and it was manifested in all dimensions of the questionnaire, was maintained when we introduced any of the variables studied and was presented in all profiles.

On the other hand, our work has revealed other variables that influence the frequency of ICT use. Thus, we have found that teachers who attend to young children use them less than teachers of older students. These data coincide with those found in other works (Gorder, 2008; Vanderlinde et al., 2010) and are probably related to the characteristics of the teaching activity itself. It is undoubtedly more arduous to use ICT in class with young children than with adolescents or adults. We have also found a greater frequency of use by generalists than specialists because the former teach more hours in the same class and consequently have more responsibilities with their students. Both the specialists and the teachers of the youngest children were overrepresented in the Passive profile. Nevertheless, the influence of the subjects taught in compulsory and non-compulsory secondary education is not so clear. We found there was hardly any influence of gender on different results. Data from other studies show that the influence of this variable is quite unstable and varies among studies (Mathews and Guarino, 2000; Gorder, 2008; Law and Chow, 2008). However, teaching experience seems to influence in another way: whereas less experienced teachers are more reproductive, the more experienced teachers present fewer differences between reproductive and constructive activities. It should be noted that in other studies this variable has also shown ambiguous results (Mathews and Guarino, 2000; Baek et al., 2008; Gorder, 2008; Inan and Lowther, 2010).

The third objective analyzed the learning outcomes that the activities provided, the type of assessment used, and the cooperation that activities promoted. In general, we have seen that teachers performed more verbal and attitudinal learning than procedural. However, in these cases (as well as in the assessment), activities were aimed at reproductive instead of constructive learning. The least frequent activities were cooperative (between never and some days per month), which is consistent with the importance given to reproduction. The salience of verbal learning increased as the higher the educational level was and, in the same way, the attitudinal activities decreased, with hardly any change in the procedural ones.

Considering that these data were collected in Spain when there were strict confinement and social isolation, we would emphasize that the activities related to attitudes were directed at maintaining classroom control in all groups and profiles (but outside the classroom) whereas there was much less frequency of activities focused on getting the ability to managing student attitudes, behavior or self-control during that situation of confinement. This difference suggests that teachers were more concerned about controlling their students’ study habits.

Regarding our fourth objective, we find four profiles of teachers (Passive, Active, Reproductive, and Interpretative). The first two differed only in the amount of total activity performed, while the Reproductive one was characterized by almost exclusively executing reproductive learning activities. Although, as in the previous groups, the Interpretative teachers carried out many reproductive activities, they also carried out constructive activities with considerable frequency. Teachers of children from 3 to 6 years, for whom engaging in the virtual activity is more complicated, abounded in the Passive profile. However, in the Reproductive profile, teachers of mathematics of secondary education predominated. In contrast, in the Interpretative profile, in which there were fewer differences between reproductive and constructive activities, generalists of primary education and teachers of social and natural sciences and Spanish language of secondary education were over-represented. But principally, this profile was over-represented by teachers who had previously used ICT.

In conclusion, it seems the teachers in this study use ICT essentially for presenting different kinds of information (Tondeur et al., 2008b) and do not use them as learning tools that help students to build, manage, and develop their knowledge. On the other hand, this study seems to show that teachers’ beliefs are much closer to the reproductive pole than to the constructive one. In this study, beliefs have been inferred through the frequency with which the teachers stated they carried out predetermined activities. In our view, the description of the activities was much closer to the actual practices and theories of the teachers than the results that questionnaires on beliefs could provide us with. For this reason, we expect the mismatch between theories and practices (Liu, 2011; Fives and Buehl, 2012; Tsai and Chai, 2012; Mama and Hennessy, 2013; Ertmer et al., 2015; de Aldama and Pozo, 2016) was smaller and helped us to discover the true beliefs of teachers when they teach.

We could therefore conclude that, despite all the educational possibilities and all the promises of change in teaching that ICT raise (Jaffee, 1997; Collins and Halverson, 2009), teachers have only perceived these tools as informative support. It seems the critical incident caused by the pandemic has not been resolved in the short-term with a change in favor of student-centered activities and content-centered ones continue predominating. Therefore, our data are more consistent with the results of some international mass studies (Biagi and Loi, 2013; OECD, 2015) than with the experimental works that analyze how teachers who are previously chosen use ICT (Tamim et al., 2011; Alrasheedi et al., 2015; Sung et al., 2015; Clark et al., 2016; Xie et al., 2018; Mayer, 2019). However, there is no doubt that the pandemic has contributed to familiarizing teachers with ICT. In our results, previous use of ICT was the variable that produced the most systematic differences in both the frequency of proposed reproductive and constructive activities. In this sense, perhaps the pandemic may have contributed to an increase in teachers’ experience in two of the three educational computer uses described by Tondeur et al. (2008a): basic computer skills and use of computers as an information tool. Maybe, this fact could contribute in the future to using the third one, the use of ICT as learning tools. However, there are undoubtedly other variables related to first-order and second-order barriers (beliefs) or teacher training with ICT that influence this possibility of change.

In summary, our work shows that activities carried out through ICT during confined schooling were more teacher-centered than student-centered and hardly promoted the 21st-century skills, that digital technologies should facilitate (Ertmer et al., 2015). However, the data also show that the greater the stated previous use of ICT, the greater and more constructive its use was reported for the pandemic. Previous use of ICTs is related not only to beliefs about their usefulness but also to specific training to master these tools and to use them in a versatile manner, adapted to different purposes or objectives. It seems clear that teacher training should be promoted not only to encourage more frequent use of ICT but also to change conceptions toward them to promote constructive learning. In this sense, the forced use of ICT because of COVID-19 will only encourage this change if we support teachers with adequate resources and activities which facilitate reflection on their use.

However, we should consider that one limitation of this study is that the practices analyzed were those declared by the teachers. It would be necessary to complete this study with an analysis of the practices that the teachers really applied and to analyze their relationship with their conceptions of learning and teaching. In fact, we are currently analyzing the actual practices of a sub-sample of the teachers who filled out the questionnaire, taking the profiles found in this work as the independent variable. In future research, it would be necessary to analyze the relationship between student learning and these different teaching practices.

## Data Availability Statement

The raw data supporting the conclusions of this article will be made available by the authors, without undue reservation.

## Ethics Statement

The studies involving human participants were reviewed and approved by the Ethics Committee of the Autonomous University of Madrid. The patients/participants provided their written informed consent to participate in this study.

## Author Contributions

J-IP: funding acquisition, project administration, conceptualiza-tion, methodology, supervision, writing – original draft, and writing – review and editing. M-PE: funding acquisition, conceptualization, methodology, validation, writing – original draft, and writing – review and editing. BC: conceptualization, methodology, data curation, formal analysis, investigation, software, writing – original draft, writing – review and editing, and visualization. DLS: conceptualization, methodology, and writing – review and editing. All authors contributed to the article and approved the submitted version.

## Conflict of Interest

The authors declare that the research was conducted in the absence of any commercial or financial relationships that could be construed as a potential conflict of interest.
